# OptImal Gamma kNife lIghTnIng sOlutioN (IGNITION) score to characterize the solution space of the Gamma Knife FIP optimizer for stereotactic radiosurgery

**DOI:** 10.1002/acm2.13936

**Published:** 2023-03-01

**Authors:** Ranjini Tolakanahalli, D Jay J. Wieczorek, Yongsook C. Lee, Martin C. Tom, Matthew D. Hall, Michael W. McDermott, Minesh P. Mehta, Rupesh Kotecha, Alonso N. Gutierrez

**Affiliations:** ^1^ Department of Radiation Oncology Miami Cancer Institute, Baptist Health South Florida Miami Florida USA; ^2^ Department of Radiation Oncology, Herbert Wertheim College of Medicine Florida International University Miami Florida USA; ^3^ Department of Neurosurgery Miami Cancer Institute, Baptist Health South Florida Miami Florida USA; ^4^ Department of Translational Medicine, Herbert Wertheim College of Medicine Florida International University Miami Florida USA

**Keywords:** Gamma Knife, inverse optimizer, lightning, stereotactic radiosurgery

## Abstract

**Objectives:**

The objective of this study is to evaluate the user‐defined optimization settings in the Fast Inverse Planning (FIP) optimizer in Leksell GammaPlan® and determine the parameters that result in the best stereotactic radiosurgery (SRS) plan quality for brain metastases, benign tumors, and arteriovenous malformations (AVMs).

**Methods:**

Thirty patients with metastases and 30 with benign lesions—vestibular schwannoma, AVMs, pituitary adenoma, and meningioma‐treated with SRS were evaluated. Each target was planned by varying the low dose (LD) and beam‐on‐time (BOT) penalties in increments of 0.1, from 0 to 1. The following plan quality metrics were recorded for each plan: Paddick conformity index (PCI), gradient index (GI), BOT, and maximum organ‐at‐risk (OAR) doses. A novel objective score matrix was calculated for each target using a linearly weighted combination of the aforementioned metrics. A histogram of optimal solutions containing the five best scores was extracted.

**Results:**

A total of 7260 plans were analyzed with 121 plans per patient for the range of LD/BOT penalties. The ranges of PCI, GI, and BOT across all metastatic lesions were 0.58–0.97, 2.1–3.8, and 8.8–238 min, respectively, and were 0.13–0.97, 2.1–3.8, and 8.8–238 min, respectively, for benign lesions. The objective score matrix showed unique optimal solutions for metastatic lesions and benign lesions. Additionally, the plan metrics of the optimal solutions were significantly improved compared to the clinical plans for metastatic lesions with equivalent metrics for all other cases.

**Conclusion:**

In this study, FIP optimizer was evaluated to determine the optimal solution space to maximize PCI and minimize GI, BOT and OAR doses simultaneously for single metastatic/benign/non‐neoplastic targets. The optimal solution chart was determined using a novel objective score which provides novice and expert planners a roadmap to generate the most optimal plans efficiently using FIP.

## 1 INTRODUCTION

Gamma Knife (GK) (Elekta AB, Stockholm, Sweden) stereotactic radiosurgery (SRS) has been commonly used in the treatment of benign and malignant intracranial tumors,^1^, ^2^, ^3^, ^4^, ^5^, ^6^ as well as various non‐neoplastic conditions, such as arteriovenous malformations^7^ and trigeminal neuralgia.^8^ The latest redesigned platforms, Perfexion™ and Icon™, consist of 8 movable sectors, each loaded with 24 Co‐60 sources, totaling 192 sources.^9^ Individually, the sectors can be set to four different positions during treatment, three defining collimator sizes of 4, 8, and 16 mm and an off (blocked) position. Thus for any given shot, there are 65 536 possible beam shapes based on the various collimator settings. As most targets have multiple shot positions, the number of possibilities increases exponentially, and therefore, if planned manually, an enormously large range of plan qualities emerge; even the experienced planner cannot realistically evaluate the optimal settings. To assist in achieving the desired dose distribution, an inverse planning tool was introduced by Elekta in the Leksell GammaPlan in 2010 that optimized coverage/selectivity, gradient index (GI) and beam‐on time (BOT) at a predefined isodose level. Obtaining an optimal plan solution, however, is inherently difficult because of the nonconvex nature of the optimization problem—difficulties arise due to the use of relative isodose lines and the variability in the shot positions.^10^

In 2020, Elekta released a new dose optimizer, Fast Inverse Planning (FIP) algorithm, commercially referred to as Lightning, which optimizes a well formulated objective function employing linear programming.^10^ The inputs fed into the optimizer for the select targets include prescription dose, maximum target dose, low‐dose (LD) penalty, BOT penalty, and maximum dose to organs‐at‐risk (OARs). The FIP algorithm addresses inverse planning in three phases: isocenter placement, optimization, and sequencing.^10^, ^11^ In the first phase, well‐distributed isocenters are generated in the target and remain fixed throughout the rest of the planning using geometrical attributes of the target. In the second phase, a cost function is formulated that maximizes dose to target while sparing OARs and minimizes BOT by combining competing objectives as a weighted sum.^10^, ^11^ High selectivity and high dose gradient are achieved by penalizing dose exceeding the prescription dose in voxels in a ring region close to the target and by penalizing dose exceeding the threshold doses in the low dose region; both the ring and the low dose regions are defined by the optimizer for single and multi‐target scenarios. During optimization, times for each sector and collimator are minimized but allowed to vary independently and are then converted to “deliverable shots” in the sequencing phase.

In our previous study, we demonstrated that plans inversely optimized using Lightning require minimal adjustments after optimization to reach target coverage and conformity goals that were clinically comparable to plans generated by expert planners, and additionally, this was achieved with significant time‐saving.^12^ The plans generated in our prior study used the default LD/BOT setting values of 0.5/0.5 (Range 0.0–1.0) with successive minimal fine tuning by the expert planner to maximize target coverage and Paddick Conformity Index (PCI). However, the inverse optimization settings, that is, range of LD/BOT penalties with or without OAR maximum dose constraint can result in a vast solution space. We define the optimal solution as the LD/BOT penalty combination which maximizes PCI and minimizes BOT, GI, and OAR max doses. This is often determined by the planner to ensure that the resulting dose distribution and metrics are acceptable for the target volume being optimized—that is, a process highly dependent on planner experience. In this study, we aim to characterize the FIP optimizer by investigating the effect of inverse optimization settings (LD/BOT) on plan quality metrics and determine the optimization parameters that result in the best SRS plan quality for both neoplastic and non‐neoplastic lesions.

## 2 METHODS

### 2.1 Case selection

After obtaining institutional review board (IRB) approval, thirty (*n* = 30) patients with single brain metastases and thirty (*n* = 30) patients with benign tumors (i.e., vestibular schwannoma (VS), pituitary adenoma (PA), and meningioma), and non‐neoplastic conditions (i.e., arteriovenous malformations [AVM]), treated with GK SRS were included in this study. The characteristics and planning directives for the target volumes in these categories are presented in Table 1.

**TABLE 1:** Distribution and volume characteristics of the 30 metastatic lesions and 30 benign and non‐neoplastic lesions investigated in this study.

	# of cases	Mean volume (cc)	Volume range (cc)	Dose (Gy)
**Benign/non‐neoplastic lesions**	**30**	2.2	0.31–8.60	
Vestibular schwannoma	9	1.1	0.31–1.78	12.5–13
Pituitary adenoma (non‐secretory)	2	1.5	0.78–1.18	16
Pituitary adenoma (secretory)	4	1.7	0.69–3.90	24
Meningioma	6	2.5	0.83–8.60	15
Arteriovenous malformation	9	3.8	0.42–6.49	18–20
**Metastatic lesions**	**30**	1.7	1.00–2.39	24

### 2.2 Inverse plan generation

The parameters that can be entered in the FIP optimizer are limited to: prescription dose, maximum target dose, LD penalty, BOT penalty, and maximum dose to OARs. In addition, a coverage option can be enabled to increase target coverage from the default of at least 95% to at least 99% when enabled. The plans included in the study were generated by providing a prescription dose, and a maximum target dose such that the prescription isodose line was greater than 50%, and with the coverage option enabled. Each target was planned by varying LD and BOT penalties in increments of 0.1 from 0 to 1 (i.e., plan #1 [0.0, 0.0], plan #2 [0.0, 0.1], …, plan #120 [1.0, 0.9], and plan #121 [1.0, 1.0]). The space encompassed by the LD/BOT combination, that is, the penalty space, resulted in 121 plans per target. Plans for all metastatic lesions (maximum dimension <2 cm) were optimized to a prescription dose of 24 Gy in a single fraction. For VS, targets were optimized to a prescription dose of either 12.5 or 13 Gy in a single fraction. Secretory PA were prescribed to 24 Gy, while the non‐secretory PA were prescribed to 16 Gy. AVMs were prescribed to 18 or 20 Gy, treated either in a single session or in a staged SRS setting. Meningiomas were prescribed to 15 Gy treated in a single fraction. Each of the 121 plans was generated by keeping the prescription dose, coverage option and maximum dose to target and OARs the same. Select plans in the benign and non‐neoplastic category that were in close proximity to OARs (brainstem, cochlea, optic apparatus, and cranial nerves) were optimized to limit maximum dose to one or more risk structures. For staged AVM cases, the treatment volume for the other stage treatments was deemed as risk volume, and maximum dose constraint was set during optimization. Metastatic lesions included in this study were not in proximity to OARs, and hence OAR dose constraints were not employed in any of the treatment plans. Additionally, to study the impact of inclusion of risk volume on the FIP plan metrics, VS cases were planned with and without the use of risk volume for the LD/BOT penalty space described above.

### 2.3 Plan quality evaluation

The following plan quality metrics were recorded for the matrix of plans generated per target volume: PCI, GI, BOT (scaled to 2.5 Gy/min output for normalization) and relevant maximum (*D*_0.03cc_) OAR doses. Each metric was reformatted into an 11 × 11 matrix (LD/BOT penalty space) such that moving from left to right increases the LD penalty along the columns while BOT penalty increases from top to bottom along the rows and where the (1,1) position holds the metric value of the plan generated with LD/BOT of 0/0 whereas the (11,11) position holds the metric value of the plan generated with LD/BOT setting of (1,1). To determine an optimal solution space that maximizes PCI, minimizes BOT and GI while meeting the OAR dose constraints, an objective score map, referred to hereafter as the opt**I**mal **G**amma k**N**ife l**I**gh**T**n**I**ng s**O**lutio**N** (IGNITION) score, was generated per plan using a weighted and scaled linear combination of the above metric matrices, that is, PCI, GI, BOT, and OAR max doses. The five plans with the lowest IGNITION scores were chosen as the optimal plans and a histogram of the optimal solution space was generated for each of the lesion categories. Metrics for the corresponding clinical plans were recorded and compared to the metrics of the extracted optimal plans, that is, the five plans with the lowest IGNITION scores.

### 2.4 IGNITION score and optimal solution set

An initial assessment of variation of PCI, GI, and BOT across the penalty space was performed that included:

1. 1D plot of BOT in an ascending order for representative plans from each category and corresponding PCI values superimposed on the same plot.
2. Pattern assessment of heat maps for individual metric matrices

Following the assessment of behavior of individual metrics, a formulation for the IGNITION score was developed and summarized in the three steps below:

1. Normalized metric matrices (i.e., PCI_norm_, GI_norm_, BOT_norm,_ and OAR_norm_) were generated by normalizing the individual metric to the maximum value in the matrix.
2. A bilinear min‐max scaling was applied to the individual elements in the above normalized matrices to generate scaled metrics (PCI_sc_, BOT_sc_, OAR_sc_, and GI_sc_). The parameters for bilinear min‐max scaling were chosen such that PCI values less than a set threshold and BOT, GI, OAR doses greater than set thresholds were penalized and scaled higher.
3. The scaled metrics 1‐PCI_sc_, BOT_sc,_, OAR_sc_, and GI_sc_ were combined into a weighted sum to generate the IGNITION score.

A short mathematical formalism that accurately represents the above description is summarized below:

Let(u,v)∈(0,0.1,..1)indicate the set of BOT and LD penalty used to generate inversely optimized plans. 1‐PCI, BOT, GI, and OAR matrices were tabulated for each of the combinations of (*u*,*v*) and represented as *H*_i_(*u*,*v*), where i∈1,2,3,4 respectively representing the above metrics. The **IGNITION score, *C*_i_(*u*,*v*)**, was then computed as a weighted linear combination of *H*’_i_(*u*,*v*), for each target and represented by Equation 1.

(1) $$C\left(u,v\right)=\sum_{i}a_{i}H{{}^{\prime }}_{i}\left(u,v\right)$$

where *H*’(*u*,*v*) = *f*(*H*(*u*,*v*)) and *a*_1_, *a*_2_, *a*_3_, and *a*_4_ represents the linear scaling for 1‐PCI, BOT, GI, and OAR metrics. The function *f*(*x*) is a bilinear min–max normalization applied to individual elements *H*(*u*,*v*) which rescales the range of features (ε_min_, ε_max_) to range in [*n*_0_, *n*]

$$f\left(x\right)=\;n_{o}+\frac{x-\varepsilon_{\min}}{\varepsilon_{\max}-\varepsilon_{\min}}\left(n-n_{0}\right)$$

The parameters used for linear weighting and min–max normalization were chosen for each category studied to mimic clinical choice; for example, parameters for GI are chosen to favor a steep dose falloff for benign lesions to minimize low dose to the surrounding OARs.

### 2.5 Statistical analysis

All data were tested for normality. A paired Wilcoxon signed‐rank test for non‐normal distributions was used to assess the statistical differences between the planning metrics, specifically, PCI, GI, and BOT for clinical and optimal FIP treatment plans. Statistical significance was established at *p* < 0.05.

## 3 RESULTS

Range of volume and size for targets in each category is shown in Table 1. For each of the 60 patients included in the study, 121 plans were generated per case resulting in a total of 7260 plans. Three metrics for each of the plans and OAR metric doses for select plans (*n* = 19) resulted in a total of 24 079 metrics which were analyzed systematically.

### 3.1 Plan quality evaluation

All plans included in the study had a minimum coverage of 99% and isodose line chosen by the optimizer was commonly 50% or greater. For metastatic lesions, range of PCI, GI, and BOT across all plans were 0.58–0.97, 2.1–3.8, and 8.8–238 min, respectively. The variation in LD/BOT penalty had a much larger impact resulting in a higher deviation about the mean for BOT (171%), followed by PCI (29%), and then GI (16%). The range of PCI, GI, and BOT across the targets included for the PA cases were 0.32–0.91, 2.3–3.9, and 10.2–380 min, respectively. The range of PCI, GI and BOT across the targets for the VS cases were 0.13–0.94, 2.3–6.7, and 6.4–175 min, respectively. The range of PCI, GI and BOT across meningioma targets were 0.28–0.93, 2.3–3.5, and 6.0–460 min, respectively. The range of PCI, GI and BOT across AVM cases were 0.35–0.92, 2.3–5.8, and 6.9–718 min, respectively. Effect of variation of LD/BOT penalty was more significant for PCI for the cases in the benign category (64%) as compared to metastases (29%). The maximum variation noted for the benign category cases about the mean for BOT was 174%, and GI was 36%.

### 3.2 IGNITION score and optimal solution histogram

**FIGURE 1:**
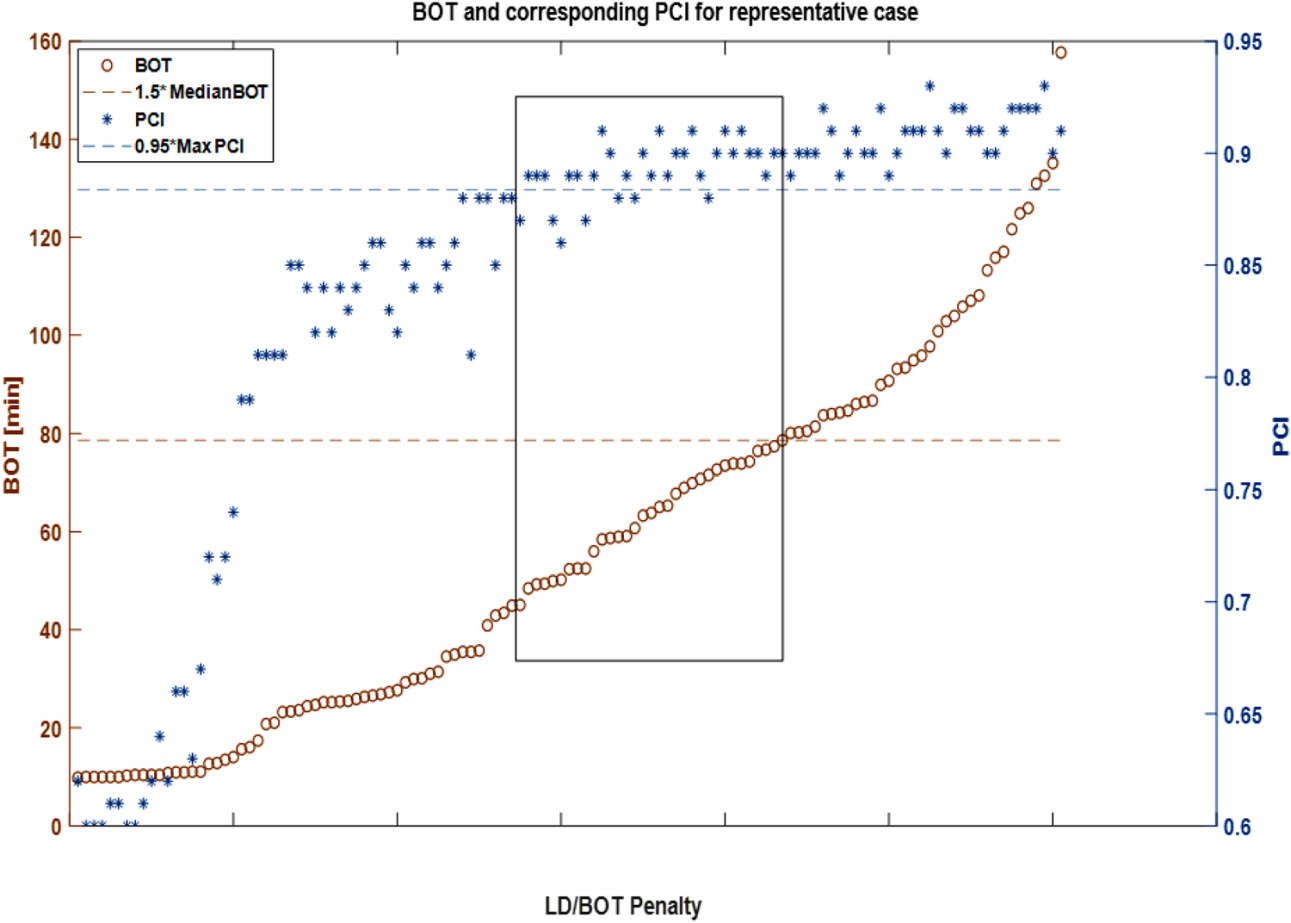
Plot of BOT (scale on the left) in increasing order is represented by brown circles and corresponding PCI superimposed on the same plot is shown with blue asterisks (Scale shown on right). The data in the rectangular block shows the optimal solution space where PCI is maximized while BOT is minimized.

**FIGURE 2:**
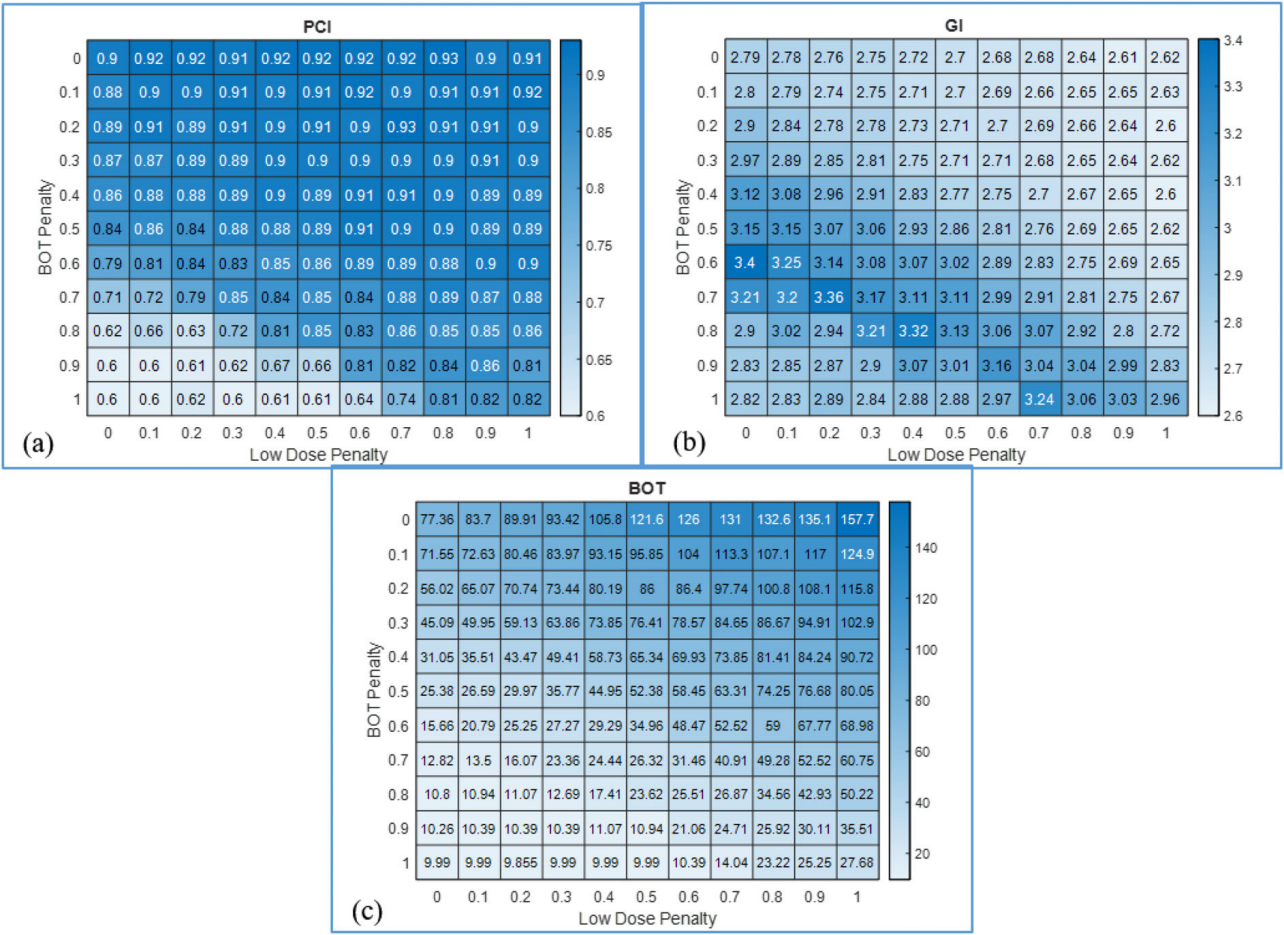
Heatmaps of PCI (a), GI (b), and BOT(c) for a representative case from the metastatic lesion category are shown.

Figure 1 shows a plot of BOT plotted in ascending order for a representative metastatic case, and the corresponding PCI was superimposed on the plot. The blue dotted line represents the threshold value of 95% of PCI. The location of the ideal plan is in the solution space as indicated between the two vertical lines where BOT is minimized and PCI is maximized. This behavior was observed across all plans included in this study. Similar behavior was observed with GI spaces when superimposed on BOT which can also be visualized in the heatmaps of PCI, GI, and BOT plotted for the representative metastatic case as shown in Figure 2a–c. The heatmaps show the calculated metrics in the penalty space with the LD/BOT setting of [0,0] occupying the top‐left corner, and the setting of [1,1] occupies the right‐lower corner. For the representative case, the optimal solution for PCI occupies the upper triangular matrix and the optimal solutions for BOT/GI occupy the lower triangular space with the overlapping space indicating the location of the optimal solution.

**FIGURE 3:**
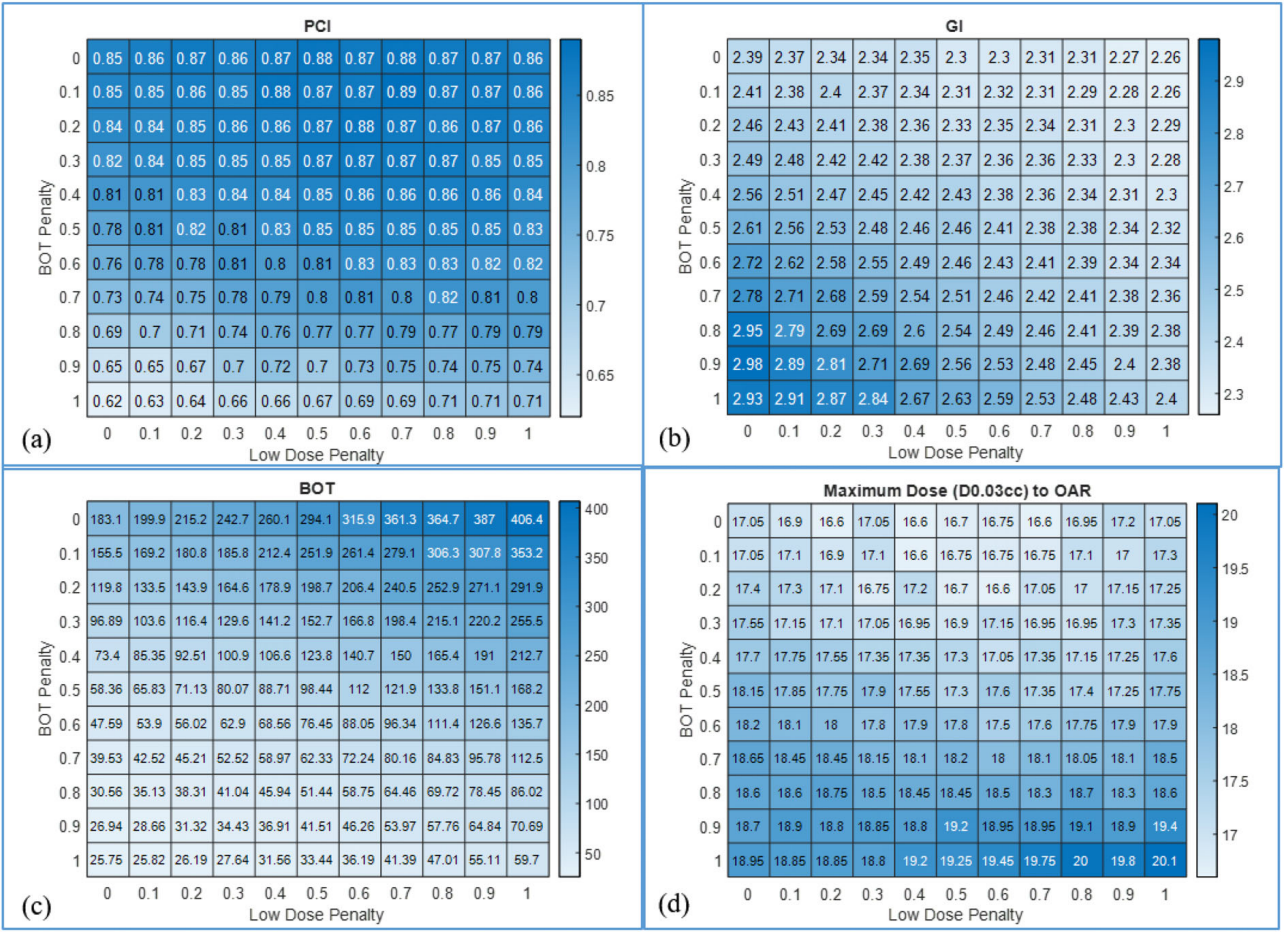
Heatmaps of PCI(a), GI(b), BOT(c), and maximum OAR doses(d) for a representative AVM case are shown.

**FIGURE 4:**
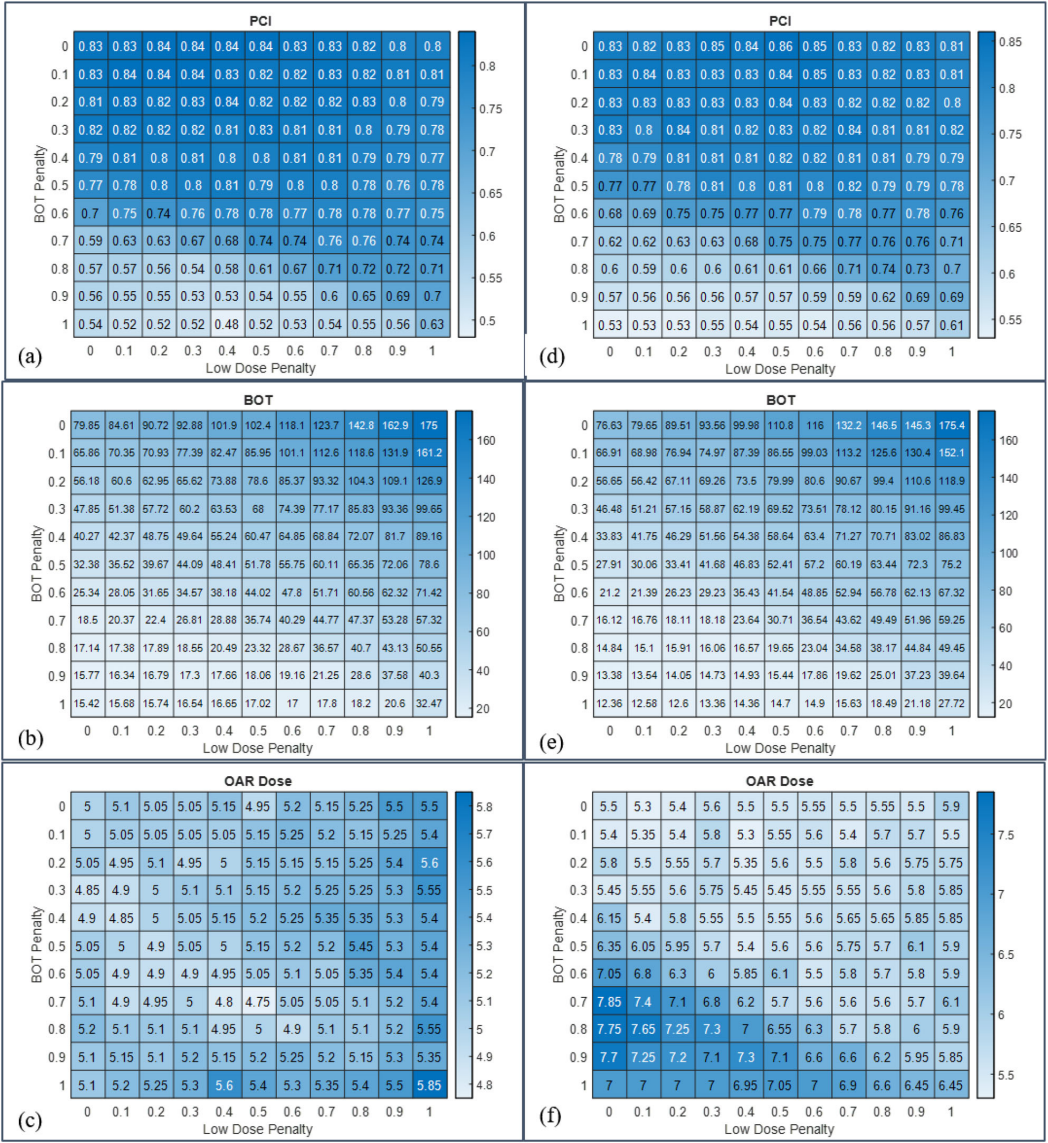
Heatmaps of PCI (a), BOT(b), and maximum dose to cochlea (c) for a representative VS case optimized with risk structure constraints are shown in the left column. The right column shows heatmaps of PCI (d), BOT(e), and max OAR dose (f) without risk structure constraints.

In Figure 3a–d, heatmaps for PCI, GI, BOT, and OAR max dose values for a representative AVM case are shown. The optimal solution for maximum OAR doses occupies the upper half of the matrix indicating that increasing BOT penalty has a higher impact on OAR dose as compared to changing the settings for the LD penalty. This is observed across all disease categories where OAR metrics were included in this study. Heatmaps for PCI and BOT of a representative VS case optimized with and without the use of OAR maximum dose constraint are shown in Figure 4a–d. Heatmaps of maximum dose to cochlea are shown in Figure 4c,f for plans optimized with and without risk structure constraint, respectively. When risk structure dose constraints are not employed, the dose to OAR increases with decreasing LD penalty and increasing BOT penalty. This is explained by the fact that as the BOT penalty is increased, the focus of the plan is to generate a shorter BOT, and hence more shots are delivered using the larger, 16 mm collimator, thereby increasing the OAR dose. Similarly, as the LD penalty is decreased, the plan is permitted to be less restrictive regarding the low dose outside the target, and hence greater dose‐spillage outside the target would raise the OAR dose.

The IGNITION score was computed by initially calculating the maximum and median values of plan metrics across 121 plans for each case followed by threshold selection for the min‐max scaling for each disease category. For metastatic lesions, PCI threshold was set to 0.95 of maximum achievable PCI for the case. PCI greater than the threshold was scaled from 0.05 to 0.1, while PCI values below 0.95 were penalized higher and scaled from 0.25 to 1.0. For VS/PA, where conformity and GI are of higher importance, PCI below the set threshold was scaled from 0.35 to 1, and GI values above the threshold of 2.8 were scaled from 0.35 to 1. The threshold for GI for the remainder of the disease categories was set from 3 to 3.2. The parameters used for bilinear normalization, and the coefficients used to compute the linear combination of 1‐PCI, BOT, and GI to generate the IGNITION score are summarized in Table 2 for each of the categories. OAR dose above the median OAR dose across all plans was scaled from 0.3 to 1 for all disease categories.

**TABLE 2:** Parameters used for the bilinear normalization used to compute the linear combination of 1‐PCI, GI, and BOT are summarized.

	1‐PCI (ε_min_, ε_max_)	1‐PCI (*n*_0_, *n*)	GI (ε_min_, ε_max_)	GI (*n*_0_, *n*)	BOT (ε_min_, ε_max_)	BOT (*n*_0_, *n*)	Weighting (1‐PCI, GI,BOT, OAR)
Vestibular schwannoma/pituitary adenoma	(0,0.05)	(0.05,0.1)	(0 2.8)	(0.05 0.1)	(0,1.5*BOT_median_)	(0 0.3)	(1.5, 0.35, 0.6, 0.35)
	(0.05,1.0)	(0.35, 1.0)	(2.8 GI_max_)	(0.35, 1.0)	(1.5*BOT_median,_ BOT_max_)	(0.3 1.0)	
Meningioma	(0,0.06)	(0.05,0.1)	(0 3.2)	(0.05 0.2)	(0,1.2*BOT_median_)	(0 0.3)	(1.0, 0.2, 0.8, 0.25)
	(0.06,1.0)	(0.25, 1.0)	(3.2 GI_max_)	(0.3, 1.0)	(1.2*BOT_median,_ BOT_max_)	(0.3 1.0)	
Arteriovenous malformation	(0,0.08)	(0.05,0.1)	(0 3.2)	(0.05 0.1)	(0,1.2*BOT_median_)	(0 0.3)	(1.0, 0.2, 1.0, 0.25)
	(0.08,1.0)	(0.25, 1.0)	(3.2 GI_max_)	(0.35, 1.0)	(1.2*BOT_median,_ BOT_max_)	(0.3 1.0)	
Metastatic lesions	(0,0.05)	(0.05,0.1)	(0 3.0)	(0.0 0.2)	(0,1.0*BOT_median_)	(0 0.2)	(1.0, 0.2, 1.0, N/A)
	(0.05,1.0)	(0.25, 1.0)	(3.0 GI_max_)	(0.3, 1.0)	(1.0*BOT_median,_ BOT_max_)	(0.3 1.0)	

To illustrate, we provide an example of a case of a metastatic lesion. Across the LD/BOT optimization parameters, the ranges of PCI, GI and BOT were [0.6 0.93], [2.6 3.4], and [9.8 157.7], respectively. We now illustrate how to compute the IGNITION score for one of the plans generated using LD/ BOT of [1,0]. For the chosen plan, the PCI, GI and BOT values were 0.91, 2.62, and 157.6 min, respectively. Normalized values of 1‐PCI, GI and BOT were 0.0215, 0.7706, and 1.0, respectively. For PCI, as shown in Table 2, since the scaled value was less than 0.05 (threshold), row 1 parameters of (ε_min_, ε_max_), and [*n*_0_, *n*] were used resulting in scaled PCI of 0.05 + (0.0215‐0)/(0.05‐0)*(0.1‐0.05) = 0.0715. Similarly, since GI was less than threshold of 3.0 (normalized value of 0.88), row 1 parameters were used which scaled the normalized GI to 0+ (0.7706‐0.7647)/(0.88‐0.7647)*(0.2‐0.0) = 0.01. The value 0.7647 used here as $\varepsilon_{min}\;$is the normalized value of minimum GI (2.6). Similarly, row2 of values were used for BOT resulting in min‐max normalized value of 1.0. Scaled addition of the three results, that is, 1*0.0715 + 0.2*0.01 + 1.0*1.0, results in a final IGNITION score for this case of 1.0735. The minimum IGNITION score for this case was 0.2994 which corresponds to a LD/BOT value of [0.7 0.8], respectively.

**FIGURE 5:**
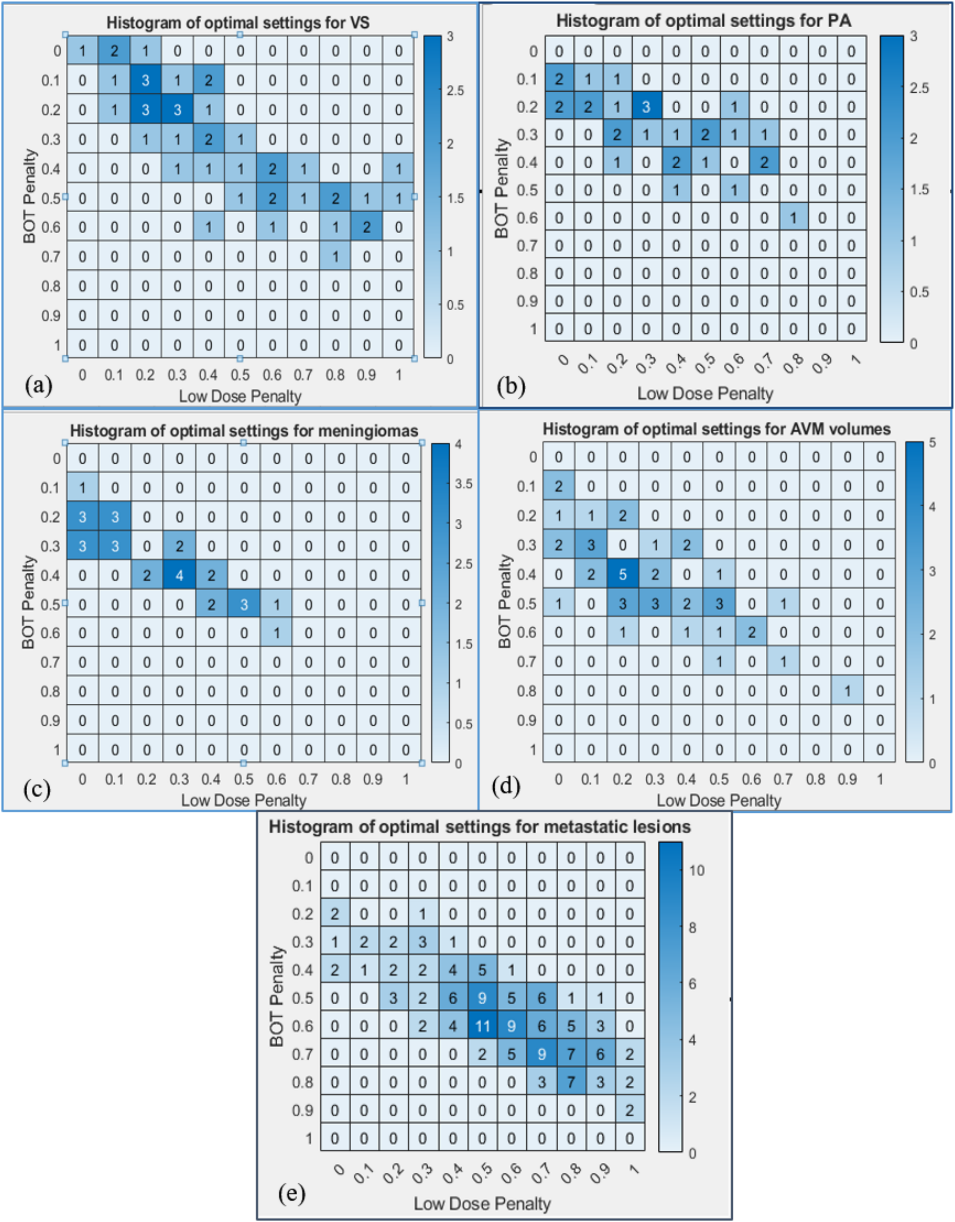
IGNITION score was calculated for each plan for all disease categories, and the optimal solution space was extracted for each case as the five plans with the lowest IGNITION score as shown in the figure. Histogram plots representing the frequency of plans with lowest ignition score for VS (a), PA (b), meningioma (c), AVM (d), and metastatic lesions (e) are shown here.

The optimal solution space was extracted for each target, utilizing the five plans (LD/BOT penalty) with the lowest IGNITION score, and the histogram plots showing these results for each of the disease categories are shown in Figure 5a–e. Figure 5 can be used as an atlas to pick optimal LD/BOT penalty per disease site to achieve optimal plan quality or to assess if the clinical plan is comparable to the optimal achievable plan.

### 3.3 Comparison with clinical data

Table 3 shows the comparison of plan metrics between the clinical plan that was actually used to deliver the treatment and median of the five optimal FIP plans for the cases included in the study. As compared to clinical plans, the FIP plans showed a statistically significant improvement in the median PCI by 1.6% (*p* < 0.01) and median GI by 2.7% (*p* < 0.01) and median BOT by 25.5% (*p* < 0.01) for metastases. The plan metrics for the average of the optimal solutions across the benign subgroups were equivalent to the expertly planned clinical cases (*p* > 0.05).

**TABLE 3:** Comparison of median plan metrics for clinical plan and optimal solution extracted using the fast inverse planning (FIP) for metastatic, benign, and non‐neoplastic lesions.

		Median PCI		Median GI		Median BOT (min)		Median OAR dose (Gy)
Vestibular schwannoma	Clinical	0.87 ± 0.08	NS	2.77 ± 0.26	NS	51 ± 17	NS	9.5 ± 3.0
	Optimal FIP	0.86 ± 0.07		2.66 ± 0.19		43 ± 16		9.3 ± 3.0
Pituitary adenoma	Clinical	0.78 ± 0.07	NS	2.7 ± 0.31	NS	100 ± 51	NS	N/A
	Optimal FIP	0.81 ± 0.05		2.7 ± 0.15		95 ± 25		
Arteriovenous malformation	Clinical	0.78 ± 0.09	NS	2.70 ± 0.20	NS	108 ± 43	NS	16.0 ± 1.7
	Optimal FIP	0.78 ± 0.06		2.54 ± 0.26		88 ± 49		12.1 ± 2.8
Meningioma	Clinical	0.84 ± 0.06	NS	2.63 ± 0.25	NS	55 ± 26	NS	N/A
	Optimal FIP	0.85 ± 0.04		2.62 ± 0.20		51 ± 25		
Metastatic lesions	Clinical	0.89 ± 0.03	*p* < 0.01	2.78 ± 0.16	*p* < 0.01	53 ± 15	*p* < 0.05	N/A
	Optimal FIP	0.90 ± 0.02		2.71 ± 0.17		40 ± 11		

## 4 DISCUSSION

Historically, treatment planning for Gamma Knife SRS used a manual forward planning technique to obtain an optimal plan that aims to maximize target coverage and selectivity, while minimizing GI and BOT. This is heavily dependent on the planner's experience and planning time available between simulation and treatment. The FIP optimizer optimizes collimator configuration and weighting in parallel for a set of well‐positioned isocenters.^11^ The solution has been designed to maximize target coverage and selectivity while minimizing BOT, GI, and maximum dose to OARs.

In our previous study, we showed that the FIP optimizer was shown to significantly reduce the efforts for treatment planning while achieving comparable plan quality of an expert planner.^12^ The FIP treatment plans were generated at a default optimization setting of 0.5/0.5 (Range: 0–1) followed by minor tweaks to the plan to achieve a target coverage of 100% and minimize PCI. Although the default optimization settings with minimal modifications produced clinically acceptable results, assessment of the inverse plan involves evaluation of dose distribution and inferring whether the resulting plan metrics, such as PCI and BOT are the most optimal solutions achievable. The LD/BOT penalty space along with the OAR maximum dose can result in a large solution space, and a systematic evaluation of the penalty space is required to assess the penalties resulting in an optimal solution. Our study quantifies the impact of LD/BOT and use of risk structure dose constraint on the performance of the optimizer, which will equip both novice and experienced planners with a solution space to choose from for each of the disease categories studied.

We noted that when BOT is sorted in an ascending order across 121 plans, it shows a quadratic trend, and the corresponding PCI shows a sharp increase, followed by saturation region. This behavior across plans provides an opportunity to extract an optimal solution space with maximum PCI and minimum BOT. This observation could also be exploited to minimize GI and OAR dose. Such a tool can serve as a “plan‐check” tool to confirm whether an individually generated plan “sits” in the optimal solution space or not, and if not, further refinements could be considered. A novel IGNITION score with a linear combination of all the plan metrics was generated for plan assessment in this study. The variation in the LD/BOT penalty had a much larger impact, resulting in a higher deviation about the mean for BOT (171%), followed by PCI (29%), and then GI (14%) for metastatic lesions while the deviation about the mean for benign and non‐neoplastic targets was BOT (194%), followed by PCI (151%), and then GI (90%).

Intuitively, one could expect that increasing the BOT penalty would decrease the BOT, and one way to achieve this is by deploying more shots using the larger (16 mm) collimators. Presumptively, this would result in decreased PCI and increased GI. Decreasing BOT penalty results in use of smaller collimators, resulting in tighter dose distribution around curved surfaces enhancing selectivity and increasing BOT. When viewed as a heatmap, the upper triangular space where LD penalty ≥ BOT penalty yields a region of maximized PCI. The lower triangular space, where BOT Penalty ≥ LD Penalty, minimizes BOT and also GI. Thus, the optimal solution space often lies around the diagonal, that is, LD penalty = BOT penalty, often with BOT Penalty < 0.8 for metastatic lesions. This relationship likely holds true for near‐spherical lesions, such as brain metastases.

In contrast, the benign and non‐neoplastic lesions were mostly irregular in shape, and were also located in close proximity to OARs requiring far more beam‐shaping. The PCI for such targets is more sensitive to increasing BOT penalty, and we observed that the optimal solutions for these lesions occupied a space with BOT penalty < 0.5. Further, for these targets the heatmap shows that increasing the BOT penalty has a much higher impact on OAR dose irrespective of LD penalty, because the increased use of 16 mm collimators results in greater OAR doses in close proximity to the target. Given the enormous planning flexibility afforded by the combination of all of the planning parameters, we observed that the use of maximum OAR dose constraint always resulted in all of the plans meeting the required constraints. As an illustrative example, in the case of VS, constraining the maximum dose to cochlea to achieve a mean dose of less than 4 Gy significantly reduced the maximum dose received by the OAR without impacting the PCI or BOT.

The IGNITION score developed to assess the metrics for this study, represents a unique way of combining multiple plan metrics across plans to extract the optimal solution with the goals of maximizing PCI, minimizing GI and providing reasonably short and clinically acceptable BOT, which a patient would be able to tolerate. This tool, primarily developed for this study, can be used to assess plan quality metrics for any future optimizers and not limited to FIP. For each case, the score was formulated to penalize metrics using bilinear min–max normalization criteria greater than set threshold criteria: a) BOT > threshold factor* BOT_median_, b) PCI < threshold factor * PCI_max_ and c) GI > threshold GI d) OAR dose > OARDose_median_ in the solution space. The threshold factors were designed to mimic clinical choice, for example, GI was penalized higher, and BOT penalized lower for irregularly shaped targets such as PA and VS cases to minimize low dose spread to the adjacent OARs, which results in some increase in BOT. The linear combination for the IGNITION score across plans weighted PCI and BOT higher, while GI and OAR doses received a lower weighting; this was designated separately for each lesion category included in the study.

Histogram analysis for VS and PA cases showed that the optimal solutions clustered around the upper diagonal space with LD ∈ (0 0.4) and BOT penalty ∈ (0.0 0.5). This clustering is a result of the score penalizing PCI and GI, while sacrificing BOT to maximize conformity and minimize low dose spread. This also results in plans preferentially employing 4 and 8 mm collimators compared to the 16 mm collimator. A comparison with the clinical plans showed that the resulting optimal solutions were comparable across all metrics for the benign lesions. In the case of AVMs, the optimal solutions clustered around the upper diagonal space. This was due to PCI being penalized higher to conform to the highly irregularly shaped and larger targets. Comparison with the clinical plans showed no deterioration in any of the plan quality metrics. The metastases included in the study, ranged in volume from 1 to 2.7 cc, were fairly regular in shape and not in proximity to any OAR. The IGNITION score showed a large solution space, with the optimal solutions clustered around the lower diagonal space, that is, LD ∈ (0.4 0.8) and BOT penalty ∈ (0.4.0 0.8). Comparison with clinical plans showed a significant improvement across all plan quality metrics.

To the authors’ knowledge, this is the first manuscript comprehensively evaluating the effect of inverse optimization settings on the clinical plan quality metrics of FIP for GK SRS treatment plans. The study does not include cases with large metastatic lesions or simultaneous optimization of multiple lesions in close proximity to each other which significantly impacts the performance of the optimizer. This is a future project that we are currently addressing. The effect of varying OAR constraints in combination with varying penalties could also result in different metrics not included in the study.

## 5 CONCLUSIONS

In this study, an analysis of the user‐inputted variables were systematically varied and a novel IGNITION score was developed to combine plan metrics to determine the optimal solution space that maximizes PCI and minimizes GI, BOT and OAR doses simultaneously for single metastatic/benign lesions and for non‐neoplastic targets. The optimal solutions extracted show equivalent metrics with expertly planned clinical cases for benign and non‐neoplastic lesions while significantly better metric values were noted for single metastatic lesions. The IGNITION score and the developed methodology and optimal solution space provides novice and expert planners a roadmap to generate the most optimal plans efficiently thus permitting for shortened time from MRI to treatment.

## AUTHOR CONTRIBUTIONS

All authors contributed to the concept development, analysis of data, initial draft of manuscript, and final approval of the manuscript. Ranjini Tolakanahalli, D Jay Wieczorek, Yongsook Lee were involved with data collection and analysis. Ranjini Tolakanahalli and Alonso Gutierrez were involved with statistical analysis of the data.

## CONFLICT OF INTEREST STATEMENT

Rupesh Kotecha: Honoraria from Accuray Inc., Elekta AB, ViewRay Inc., Novocure Inc., Elsevier Inc., and Brainlab and institutional research funding from Medtronic Inc., Blue Earth Diagnostics Ltd., Novocure Inc., GT Medical Technologies, AstraZeneca, Exelixis, ViewRay Inc., and Brainlab. Matthew D Hall: Proton Collaborative Group Executive Committee Institutional Representative and Voting Member, Miami Cancer Institute (unpaid). Grant Funding: Live Like Bella Pediatric Cancer Research Initiative, Florida Department of Health Grants 8LA04 and 22L01. Michael W McDermott: Alonso N Gutierrez: Honoraria from ViewRay, Inc., Elekta AB, IBA AB. Minesh P Mehta: Consulting Fees from Karyopharm, Sapience, Zap, Mevion, Xoft; BOD Oncoceutics; Stock in Chimerix. Martin C Tom: Honoraria from ViewRay. Institutional research funding from Blue Earth Diagnostics. Ltd. Personal fees from Elsevier. All remaining authors declare no conflicts of interest.

## Data Availability

“Research data are not available at this time.”
